# Characterization of the Antinociceptive Mechanisms of Khat Extract (*Catha edulis*) in Mice

**DOI:** 10.3389/fneur.2017.00069

**Published:** 2017-03-02

**Authors:** Elham A. Afify, Huda M. Alkreathy, Ahmed S. Ali, Hassan A. Alfaifi, Lateef M. Khan

**Affiliations:** ^1^Faculty of Pharmacy, Alexandria University, Alexandria, Egypt; ^2^Department of Pharmacology and Toxicology, Faculty of Pharmacy, King Abdulaziz University, Jeddah, Saudi Arabia; ^3^Faculty of Medicine, Department of Pharmacology, King Abdulaziz University, Jeddah, Saudi Arabia

**Keywords:** khat (*Catha edulis*), hot plate, formalin, tail flick, opioidergic, dopaminergic, GABAergic

## Abstract

This study investigated the antinociceptive mechanisms of khat extract (100, 200, and 400 mg/kg, i.p.) in four pain models: two thermic (hot plate, tail-flick) and two chemical (acetic acid, formalin) models. Male mice were pretreated intraperitoneally (i.p.) with the opioid receptor blocker naloxone (5 mg/kg), the cholinergic antagonist atropine (2 mg/kg), the selective α_1_ blocker prazosin (1 mg/kg), the dopamine D_2_ antagonist haloperidol (1.5 mg/kg), or the GABA_A_ receptor antagonist, bicuculline (1 mg/kg) 15 minutes prior to i.p. injection of khat extract (400 mg/kg). Khat extract reduced the nociceptive response of mice in the four pain tests. Naloxone significantly inhibited the antinociceptive effect of khat extract in the hot plate, tail-flick, and the first phase of formalin tests. Bicuculline significantly antagonized the antinociceptive effect of khat extract on the hot plate and tail-flick tests. Haloperidol significantly reversed the antinociceptive effect of khat extract on the tail-flick test and the first phase of formalin test. These results provide strong evidence that the antinociceptive activity of khat extract is mediated *via* opioidergic, GABAergic, and dopaminergic pathways. The mechanism of the antinociceptive action of khat may be linked to the different types of pain generated in animal models.

## Introduction

Pain is a common reason to seek medical consultation due to its high prevalence (1). Both short-term and chronic use of most of the current pain medications such as NSAIDs and opioids is associated with many adverse effects and potential interactions with other medications (2). This explains the increasing interest in the use of herbal medicine as an alternative or adjuvant for classic analgesics (3). Khat (*Catha edulis*) is a medium-sized evergreen tree of psychostimulant nature that is cultivated in Yemen and East African countries (4). Chewing of khat leaves is a traditional habit in different populations to alleviate fatigue and produce analgesia (5–7). Detailed description of chemical and pharmacological profile and negative health impact of khat abuse were documented and critically reviewed (8, 9).

Among 20 psychoactive substances, khat is considered as the least harmful herb of low dependence potential (10). The major active constituents of khat extract include six major alkaloids, tannins, and flavonoids (11). The WHO recommended that the potential for abuse and dependence of khat and its constituents is low and that its impact on public health do not warrant international control (12). Moreover, khat has some positive characteristics, which encourage its further studies. These effects include lowering of plasma cholesterol and reduction in glucose and triglycerides concentration (13) in addition to its potent cytotoxicity and antibacterial activity (14). These effects are predominantly due to cathinone, the main psychoactive component in khat. Interestingly, the use of khat could be beneficial in pain management due to the poor abuse liability of the active constituents. Earlier studies have demonstrated prolonged analgesic effect induced by cathinone (15) and khat in animal models (16, 17). Both opiate (16) and non-opiate (15, 18) pathways have been suggested as a mechanism for the antinociceptive action of khat. However, the identification of the exact receptors mediating the antinociceptive action of khat is not investigated.

The process of pain is a complex phenomenon that is connected to the type of the painful stimulus, the type of nerves involved in pain transmission, and the neurotransmitters released (19). The link between the antinociceptive effect of khat and the type of pain elicited by the nociceptive signal has not been addressed. Different nociceptive stimuli including thermal, chemical, and inflammatory signals activate specific types of nerve fibers, namely, Aδ and C fibers (20, 21). The difference in the mechanism of action of analgesic agents as opioids based on the pain test has been documented (22). Although numerous studies have investigated the antinociceptive effect of cathinone, only few studies have dealt with the effects of whole material, as normally taken by users and none of these studies addressed the link between the antinociceptive effect of khat and the type of painful stimulus and if there is a direct evidence of the involvement of different neurotransmitters in the antinociceptive action of khat following acute administration. In this study, we investigated the antinociceptive mechanisms of khat extract in relation to the type of pain tested. Two chemical pain tests, acetic acid-induced writhing and formalin-induced licking in addition to two thermal models, hot plate and tail-flick tests were performed on mice. The involvement of different receptors in the antinociceptive action of khat was also investigated using the opioid antagonist naloxone, the cholinergic antagonist atropine, the selective α_1_ blocker prazosin, the dopamine D_2_ antagonist haloperidol, and the GABA_A_ receptor antagonist, bicuculline.

## Materials and Methods

### Animals

Male Swiss mice (30–40 g) from King Fahd Medical Research Center, King Abdulaziz University, Jeddah, Saudi Arabia were used in the present study. The mice were kept in cages under standardized conditions with 12/12 h light/dark cycle in a temperature-controlled room at 22 ± 2°C with free access to food and water. The study was performed according to the institutional recommendations for the care and use of experimental animals of the research and ethics committee, ethical approval No. 109/1438/BR. The protocol was approved by the research ethical committee, substance of abuse research center. All animals were used only for one procedure and were humanely sacrificed under anesthesia after the completion of experiment. The experiments were performed by an observer blind to the treatment type.

### Drugs

Morphine sulfate, diclofenac sodium, and haloperidol were from Jamjoom Pharma Factory (Jeddah, KSA). Bicuculline and atropine sulfate were from Sigma Chemical Co. (St. Louis, MO, USA). Prazosin and naloxone were from Tabuk Pharmaceuticals Manufacturing Company (Kingdom of Saudi Arabia). Atropine sulfate, morphine, and naloxone were dissolved in saline. Bicuculline was dissolved in normal saline with few drops of concentrated acetic acid 97% (23). Haloperidol ampoule (5 mg/mL) was diluted in saline.

### Preparation of Khat Extract

A total of 300 g of fresh plants of *C. edulis* (stem tips and leaves) were provided by Substance Abuse Research Center (Jizan University). The transported fresh packed bundles were carried on the same day in an ice box to the laboratory and stored at −20°C overnight. The dried plants were washed with distilled water to remove dust and debris and dried in freeze dryer for two nights. The dried leaves (weighed 88 g) were crushed with pestle in a mortar and immersed with ethanol 96% in a flask (24). The mixture of khat material and ethanol was stirred gently and then left to stand overnight wrapped with aluminum foil to avoid light-induced decomposition. The content was later filtered, first by use of gauze roll to separate the big particles followed by filter paper to remove the fine particles (25). The filtrate was evaporated under rotary pump to remove ethanol then, in a vacuum at 40°C to remove all traces of ethanol. The resulting ethanol-free extract (12 g) constituted 4% of the original fresh material (26). Extracts were stored in refrigerator after wrapping in parafilm. The batch of 300 g of fresh leaves bundles produced 12 g of dry extract representing 100:4 leaf-to-extract extraction ratio. Khat extract was freshly dissolved in normal saline and kept in refrigerator at 4°C prior to use. The identity of the active constituents was confirmed by mass spectroscopy and showed that both cathinone and cathine existed in approximately equal concentration.

### Mass Spectra

**Figure 1:**
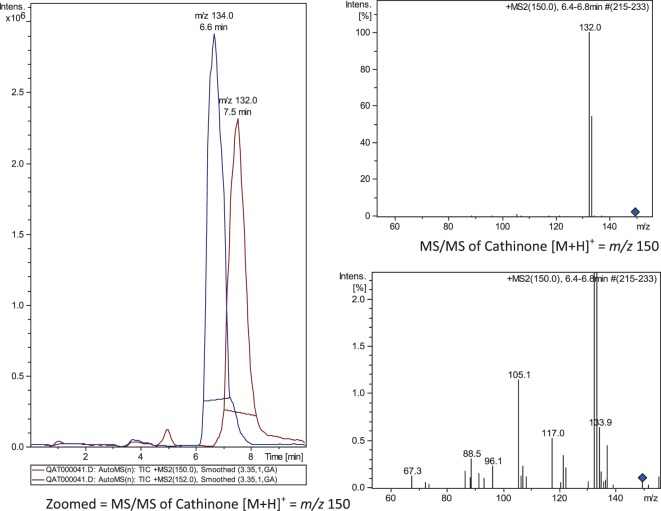
**MS/MS chromatogram (MS2) of *m*/*z* 150 (→ 132) and 152 (→ 134)**. Cathine, at 6.6 min; cathinone at 7.5 min.

A weight of 20 mg of sample powder was extracted by 5 mL of methanol, filtered through 0.45 μm nylon filter, dried with nitrogen gas, and the residue was reconstituted in 50 μL methanol. A volume of 5 μL was injected for LC-MS analysis (Agilant 6300 Ion trap, USA) applying positive-Auto-MSn mode. A representative MS chromatogram (extracted ion chromatogram) showed cathinone *m*/*z* 134 at 6.6 min and cathine *m*/*z* 132 at 7.5 min (Figure 1). The average MS spectra were confirmed by NIST2008 database.

### Experimental Groups and Protocols

#### Animal Testing

##### Acetic Acid-Induced Abdominal Writhing

The experimental groups of mice (*n* = 6) were treated with khat extract (i.p.) 30 min before the administration of 0.6% acetic acid solution (10 mL/kg, i.p.). Injection of acetic acid in mice showed characteristic abdominal constrictions in the form of muscle contraction joined with hind limb stretching (27). The nociception intensity was quantified by counting the total number of writhings within 30 min after acetic acid injection.

##### Formalin-Induced Pain

The animals were treated with 50 μL of 1% formalin in region of the right hind paw. Following formalin injection, the mouse injected paw was observed for 30 min in an acrylic box. The time for licking the paw was observed in two phases, 0–5 min (neurogenic pain), and 15–30 min (inflammatory pain) (28).

##### Hot Plate Test

Each mouse was placed on a metallic surface of the hot plate (Ugo Basile Comerio, Italy) kept at 50 ± 1°C. After 30 min of injections of the animals, the latency in seconds was recorded using stop watch as the time between adjusting the animal on the hot plate and the appearance of symptoms of discomfort as licking of the hind paws, shaking, or jumping off from the surface. The cutoff time of 60 s was selected according to Woolfe and MacDonald (29).

##### Tail-Flick Test

A radiant heat analgesiometer (Ugo Basile Tail Flick Apparatus, Comerio, Italy) was used as described by D’Amour and Smith (30). The light beam was focused on the mouse’s tail from above after wrapping the animal gently in a cotton towel. The recorded response was a tail flick away from a light source. A stopwatch was used to measure the reaction time to the nearest second. The intensity of radiant heat was adjusted to yield the baseline latencies of 6–8 s. Under the current experimental conditions, the cutoff time was 15 s. This long cutoff time was imposed to reduce the intensity of the stimulus as a protection against tissue damage since the test was repeated every 10 min as reported earlier by Connor et al. (17). Each mouse was tested twice, before drug or vehicle administration and 10, 20, and 30 min thereafter.

#### Effect of Khat Extract on Pain Perception in Pain Models

For each pain test, a total of five groups of male mice (*n* = 6–8) was used and received the following treatments, group I: vehicle (saline) served as control. The second, third, and fourth groups were treated with three doses of khat extract, 100, 200, and 400 mg/kg i.p., respectively. The fifth group (positive control) injected with morphine (1 mg/kg, i.p.) in case of hot plate and tail-flick tests or diclofenac sodium (20 mg/kg, i.p.) in case of formalin test and acetic acid-induced writhing. In pilot experiments, lower doses of khat extract 25 and 50 mg/kg are used. Only 50 mg/kg produced slight inhibition in the acetic acid-induced writhing. These lower doses levels were also tested in other behavioral tests performed as locomotion and sedative activity and found to be ineffective. Therefore, we selected three dose levels to perform the antinociception experiments based on trials and on a previously published study (31), as well as considering the estimated amount of khat weight administered by chewers and the yield of khat extract. The responses were recorded for 30 min after khat treatment according to the test performed as previously mentioned under Section “Experimental Groups and Protocols.” The use of positive control either morphine or diclofenac was based on the mechanism of the pain test. For testing the mechanisms of analgesia as in acetic acid-induced writhing and formalin test, diclofenac is used. Morphine is used as a positive control to test analgesia due to inhibition of centrally transmitted pain as in the tail-flick test and hot plate test. The time was fixed for 30 min in all pain tests for the comparative purposes and to minimize pain sensation in animals from repeated exposure to painful stimuli as heat. Similar time protocol was previously utilized (17).

#### Characterization of Antinociceptive Mechanisms of Khat Extract

Experiments were designed to elucidate possible mechanisms by which khat extract induced antinociception in mice in the four pain models. For each pain test, mice were divided into seven groups. Since the 400 mg/kg dose of khat extract produced maximal antinociceptive effect in the four pain models, we decided to test this dose further to study the mechanism of action of khat. Group I: (control) received equal volumes of saline. Group II: khat extract (400 mg/kg, i.p.), Group III: opioid receptor antagonist naloxone (5 mg/kg, i.p.), Group IV: the cholinergic antagonist atropine (2 mg/kg, i.p.), Group V: the selective α_1_ blocker prazosin (1 mg/kg, i.p.), Group VI: the dopamine D_2_ antagonist haloperidol (1.5 mg/kg, i.p.), and Group VII: the GABA_A_ receptor antagonist, bicuculline (1 mg/kg, i.p.). The doses of the blockers used were based on pilot experiments and literature review (32) to block the receptors. These doses are used in the four pain tests and are kept constant for the comparative purposes. All blockers treatment were done 15 min prior to i.p. administration of 400 mg/kg khat extract (Groups III–VII). According to the test performed, the response related to the pain test was measured for 30 min after khat treatment as previously described under Section “Experimental Groups and Protocols.”

### Statistical Analysis

Results are presented as mean ± SEM for each experimental group. Statistical comparison of the data was performed by one-way analysis of variance followed by Bonferroni’s *post hoc* test using the GraphPad Prism software (version 5.0) for Windows. The level of significance was set at *P* < 0.05.

## Results

### Khat Extract Produced Antinociception in All Models of Pain

#### Hot Plate Test

**Figure 2:**
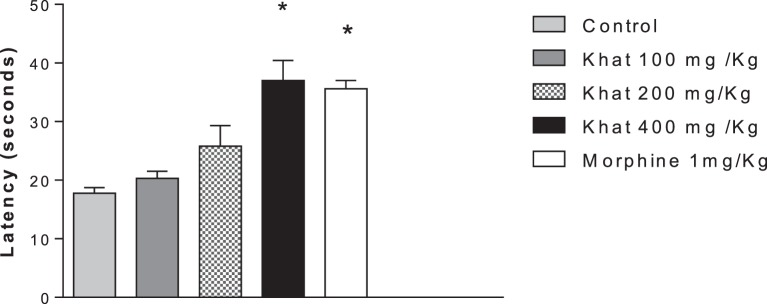
**Effect of khat extract (100, 200, and 400 mg/kg ip.), morphine (1 mg/kg ip.) on the nociceptive response of mice in the hot plate**. Values are mean ± SEM of the latency for the nociceptive behavior (seconds) (*n* = 6–8). Significantly different from control group at *P < 0.05 (*F* value = 10.991, ANOVA followed by Bonferroni’s *post hoc* test).

Treatment of mice with khat extract for 30 min significantly increased the latency only with 400 mg/kg of khat extract compared to the control value of 17.75 ± 0.95 s. The positive control morphine (1 mg/kg i.p.) produced a significant change in the latency 37.33 ± 1.42 (Figure 2).

#### Tail-Flick Test

**Figure 3:**
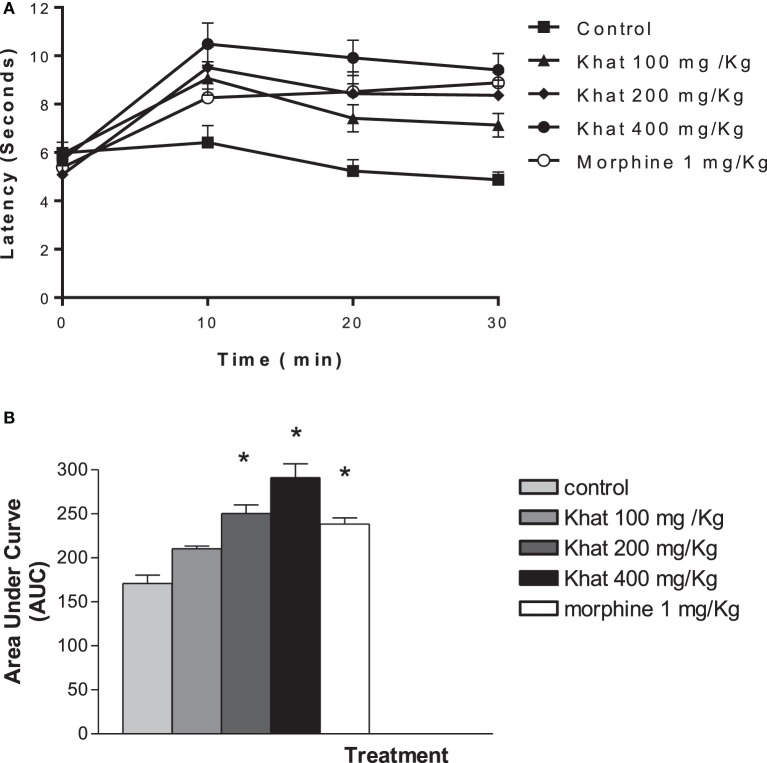
**Time course of khat extract (100, 200, and 400 mg/kg i.p.) and morphine (1 mg/kg, ip) in the mouse tail-flick test (A)**. The lower panel **(B)** shows the area under curve (AUC) from 0 to 30 min. Values are mean ± SEM (*n* = 6–8). Significantly different from control group at *P < 0.05 (*F* value = 46.997, ANOVA followed by Bonferroni’s *post hoc* test).

Compared with the control mice, i.p., administration of khat extract (200 and 400 mg/kg) resulted in a significant enhancement of the tail-flick latency. The antinociceptive effect of the intermediate and high doses of khat extract was superior than 1 mg/kg, i.p. morphine. Khat extract (400 mg/kg, i.p.) seemed to be most active; the peak was achieved at 10 min (10.48 ± 0.87 vs. 6.41 ± 0.70 min), time to tail flick was doubled at 30 min post-injection (9.41 ± 0.67 vs. 4.87 ± 0.31 min). Transforming data to area under curve scores indicated dose-dependent antionciceptive effect of khat extract (Figure 3).

#### Acetic Acid-Induced Abdominal Writhing

**Figure 4:**
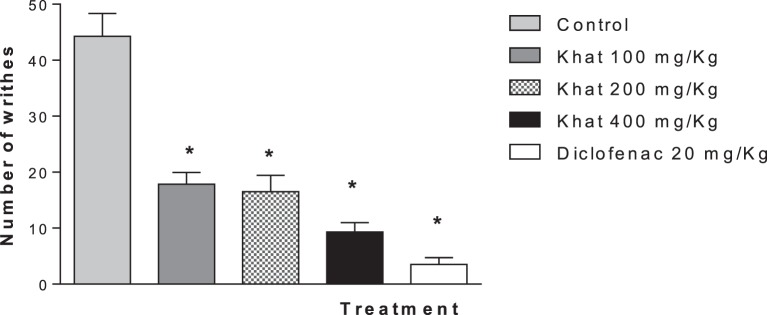
**Effect of khat extract (100, 200, and 400 mg/kg i.p.) on the number of acetic acid-induced writhes in mice**. Diclofenac (20 mg/kg i.p.) was used as positive control. Values are mean of cumulated writhings in 30 min ± SEM (*n* = 6–8). Significantly different from control group at *P < 0.05 (*F* value = 35.16, ANOVA followed by Bonferroni’s *post hoc* test).

The injection of 0.6% acetic acid solution (0.1 mL/10 g) to mice induced a characteristic writhing response between 0 and 30 min later, which was significantly (*P* < 0.05) inhibited by pretreatment with diclofenac (20 mg/kg). Khat extract (100, 200, and 400 mg/kg, i.p.) injected 30 min prior to the stimulus injection caused dose-dependent inhibition of the abdominal constrictions (59.7–78.9%; *P* < 0.05) compared with the control group (Figure 4). The positive control group produced 92% inhibition.

#### Formalin test

**Figure 5:**
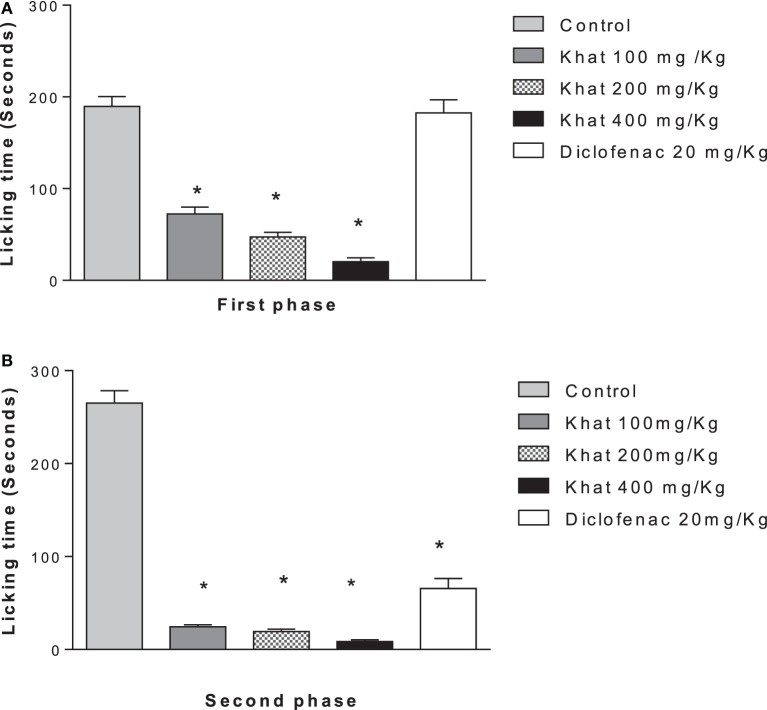
**Effect khat extract (100, 200, and 400 mg/kg i.p.), diclofenac (20 mg/kg i.p.) on the licking time in seconds of formalin-induced pain in mice, in the first phase (A) (0–5 min), *F* value = 70.030 and the second phase (B) (15–30 min)**. Values are mean ± SEM (*n* = 6–8). Significantly different from control group at *P < 0.05 (*F* value = 170.87, ANOVA followed by Bonferroni’s *post hoc* test).

Treatment of animals with khat extract (100, 200, and 400 mg/kg, i.p.) or diclofenac 20 mg/kg i.p. reduced the licking time of hind paw by 61.8, 75, 89.2, and 3.6%, respectively, in the first phase (0–5 min) vs. control value of 189.5 ± 10.89 s. In the inflammatory phase (15–30 min), treatment with khat extract (100, 200, and 400 mg/kg, i.p.) or diclofenac 20 mg/kg i.p. reduced the licking time by 90, 92.4, 96.6, and 74.3%, respectively, from a control value of 265.12 ± 13.12 s (Figures 5A,B).

### Naloxone, Bicuculline, and Haloperidol Antagonized the Antinociceptive Effect of Khat Extract

**Figure 6:**
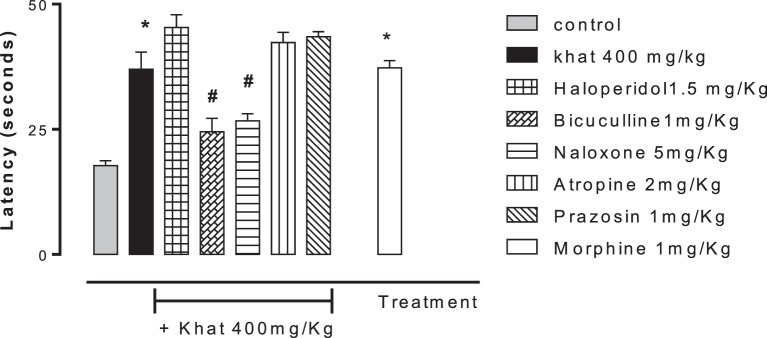
**Effect of haloperidol (1.5 mg/kg, i.p.), bicuculline (1 mg/kg, i.p.), naloxone (5 mg/kg, i.p.), atropine (2 mg/kg, i.p.), and prazosin (1 mg/kg, i.p.) on antinociception induced by khat extract (400 mg/kg, i.p.) on the hot plate latency in mice**. Drugs or saline (control) were administered 15 min prior to khat extract. Values are mean ± SEM (*n* = 6–8). Significantly different from control group at *P < 0.05. Significantly different from khat group at ^#^*P* < 0.05 (*F* value = 28.594, ANOVA followed by Bonferroni’s *post hoc* test).

**Figure 7:**
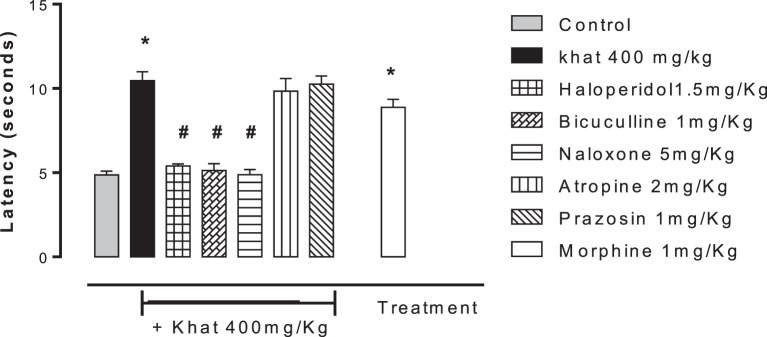
**Effect of haloperidol (1.5 mg/kg, i.p.), bicuculline (1 mg/kg, i.p.), naloxone (5 mg/kg, i.p.), atropine (2 mg/kg, i.p.), and prazosin (1 mg/kg, i.p.) on antinociception caused by khat extract (400 mg/kg, i.p.) on the tail-flick test in mice**. Drugs or saline (control) were administered 15 min prior to khat extract. Values are mean ± SEM (*n* = 5–8). Significantly different from control group at *P < 0.05. Significantly different from khat group at ^#^*P* < 0.05 (*F* value = 32.140, ANOVA followed by Bonferroni’s *post hoc* test).

**Figure 8:**
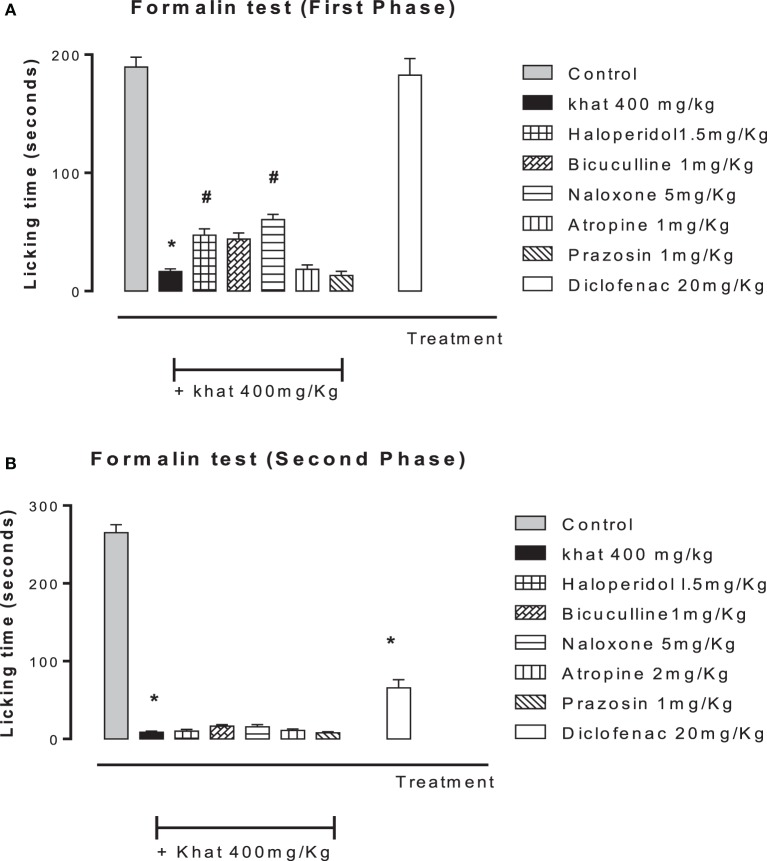
**Effect of haloperidol (1.5 mg/kg, i.p.), bicuculline (1 mg/kg, i.p.), naloxone (5 mg/kg, i.p.), atropine (2 mg/kg, i.p.), prazosin (1 mg/kg, i.p.) on antinociception induced by khat extract (400 mg/kg, i.p.) on the licking time of formalin-induced pain in mice, in the first phase (A) (0–5 min) *F* value = 96.059 and the second phase (B) (15–30 min)**. Drugs or saline (control) were administered 15 min prior to khat extract. Values are mean ± SEM (*n* = 5–8). Significantly different from control group at *P < 0.05. Significantly different from khat group at ^#^*P* < 0.05 (*F* value = 196.87, ANOVA followed by Bonferroni’s *post hoc* test).

**Figure 9:**
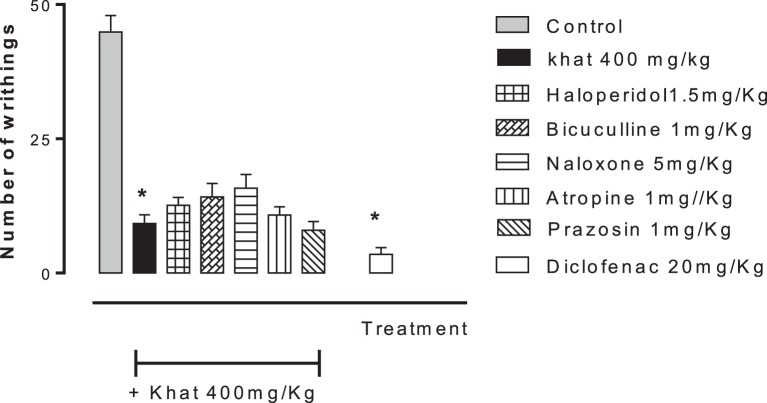
**Effect of haloperidol (1.5 mg/kg, i.p.), bicuculline (1 mg/kg, i.p.), naloxone (5 mg/kg, i.p.), atropine (2 mg/kg, i.p.), and prazosin (1 mg/kg, i.p.) on antinociception induced by khat extract (400 mg/kg, i.p.) in acetic acid-induced writhing in mice**. Drugs or saline (control) were administered 15 min prior to khat extract. Values are mean ± SEM (*n* = 5–8). Significantly different from control group at *P < 0.05. Significantly different from khat group at ^#^*P* < 0.05 (*F* value = 44.136, ANOVA followed by Bonferroni’s *post hoc* test).

None of the blockers tested changed the nociceptive response of mice in the four animal models or produced analgesia *per se* compared to the control group (data are not shown). Khat extract (400 mg/kg, i.p.) significantly increased the pain latency in *the hot plate test*. The nociceptive threshold was increased by 2.2-fold compared to the control value, 37 ± 3.43 vs. 17.75 ± 0.95 s, respectively. The antinociceptive effect of khat extract was reversed by prior administration of the opioid receptor antagonist naloxone and the GABA_A_ antagonist bicuculine but unaffected by the centrally acting dopamine D_2_-receptor antagonist, haloperidol, the α_1_ blocker prazosin or the cholinergic blocker atropine (Figure 6). In the *tail-flick test*, prior administration of naloxone, bicuculline, or haloperidol inhibited the antinociceptive effect of khat extract (Figure 7). On the other hand, the antinociceptive effect of khat extract was reversed by prior administration of naloxone or haloperidol during the *first phase of formalin test* (Figure 8A). None of the blockers tested significantly changed the licking time during the second phase of formalin test (Figure 8B) or the number of writhes induced by acetic acid administration (Figure 9).

## Discussion

In this study, khat extract markedly increased the pain response in two thermally induced pain models, namely, the hot plate and tail-flick tests and inhibited the chemically generated writhing and licking responses induced by acetic acid and formalin injection, respectively. The opioidergic, GABAergic, and dopaminergic receptors but not the adrenergic or muscarinic ones are involved in the antinociceptive action of khat. The opioidergic and dopaminergic mechanisms are involved in the antinociceptive action of khat probably involving Aδ fibers. This is the first study to link the antinociceptive action of khat to particular pain fibers. Khat extract and its constituent cathinone have been shown to have analgesic properties in similar pain tests (17, 33, 34). In addition, cathine (Norpseudoephedrine), a metabolite of cathinone enhanced the analgesic effects of morphine in hot plate and formalin tests in mice (34). Thus, our results confirmed previous reports that khat extract attenuated pain signals generated by different painful stimuli.

The present results demonstrated that the opioid antagonist, naloxone, attenuated the antinocieptive response of khat extract in hot plate, tail-flick, and first phase of formalin tests. This is in agreement with previous results (18, 33, 34) reported that cathinone-induced analgesia was antagonized by naloxone pretreatment. In contrast, our results indicated that naloxone pretreatment has no effect on the analgesic effect of khat in mice challenged with acetic acid and during the second phase of formalin test. The biphasic action of formalin has different nociceptive pathways. The first phase (neurogenic) is detected by central nociceptive afferent terminals stimulating the Aδ fibers. The second phase is an inflammatory response due to direct stimulation of chemical nociceptors resulting in an increased input from C fibers (28). Similarly, visceral pain induced by acetic acid is perceived *via* C fibers (27). The painful thermic stimuli in hot plate and tail-flick tests are also involving stimulation of the Aδ fibers. It has been shown that the opioid antagonists naloxonazine and naltriben differently attenuated antinociception involving C fibers compared with Aδ fiber-mediated response (35). Thus, it is conceivable that naloxone inhibited the analgesic effect of khat extract in pain tests mediated *via* Aδ fibers, namely, hot plate, tail-flick, and first phase of formalin test but not those activating the C fibers as second phase of formalin test and acetic acid-induced writhing. The differential antinociceptive effect for analgesic agents according to the painful stimulus was reported earlier for khat (17), morphine (22, 36), and for other centrally acting drugs such as amphetamine (18) and nicotine (37). Taken together, the opioidergic mechanism is involved in the antinociceptive action of khat probably associated with Aδ fibers.

Pretreatment of mice with bicuculline attenuated the antinociceptive effect of khat extract in hot plate and tail-flick tests. Reports about the involvement of GABA in khat analgesia are rare. One study reported that prolonged khat administration resulted in excessive release followed by decreased GABA concentration in different brain areas (38). Since bicuculline is a competitive antagonist of GABA_A_ receptor, the behavioral effects of khat extract following bicuculline administration can only be due to reversal of GABA-mediated analgesia. Thus, it could be speculated that the acute khat consumption adopted in our study increased GABA level in brain as previously detected in the early phase of chronic khat habituation (39). Therefore, the GABA_A_ antagonist, bicuculline, attenuated the antinociceptive action of khat extract in hot plate and tail-flick tests.

Interestingly, haloperidol, the D_2_-receptor antagonist, reversed the antinociceptive effect of khat extract in the tail-flick test and the first phase of formalin test, but not the hot plate test, second phase of the formalin test, and acetic acid test. As reported earlier, haloperidol did not inhibit cathinone analgesia in acetic acid-induced writhing (33). Cathinone increased the release of dopamine from presynaptic sites and increased extracellular dopamine concentration (40, 41). Both hot plate and tail-flick tests are thermic stimuli involving stimulation of Aδ fibers; yet, they follow different neural pathways. The tail flick is a spinal reflex, whereas the hot plate response is under supraspinal control (36, 37, 42, 43). It is a reasonable question to query whether the antagonistic effect of haloperidol on khat analgesia in the tail-flick test is mediated spinally through the dopaminergic pathway and not supraspinally as in the hot plate test. Since our results indicated that khat abolished all types of painful sensations, the analgesic effect produced by systemically administered khat occurs because of the potentiated interaction that exists between spinal and supraspinal effects achieved simultaneously suggesting a cross-talk between the two pathways. Thus, the results in our hand only excludes a specific role of dopamine in mediating the effect of khat extract on pain signals induced in hot plate test or those initiated *via* stimulation of C fibers following acetic acid and formalin administration.

One may argue that the results of antinociceptive action of khat could be attributed to the sedative action or the effect of the khat extract on the locomotor activity. In fact, cathinone administration in rats (44, 45) and mice (46) markedly increased spontaneous locomotor activity almost comparable with amphetamine (47). Similar increases in locomotor activity were observed after acute and subchronic oral administration of *C. edulis* leaves or cathinone in rats (48, 49). Moreover, both antinociception and locomotion are mediated *via* different pathways. Reports indicated that the potent antinociceptive action of morphine is associated with increased locomotor activity in mice (50). This confirms that the observed antinociceptive action of khat extract in the pain tests is not attributed to the effect on motor activity.

Our results indicated that the muscarinic antagonist atropine and the selective α_1_ blocker prazosin did not modify the analgesic effect of khat extract. This is the first study to address whether cholinergic or α1 receptors are involved in khat analgesia. Agonists of cholinergic and adrenergic systems are targets for the treatment of pain (51). Previous studies have shown that the non-selective α blocker phenoxybenzamine reversed cathinone-induced analgesia (33). The vasoconstrictor effect of khat on coronary vessels was independent of α_1_ adrenergic receptor stimulation (52). In a randomized clinical study, it was found that the α_1_ receptors are not involved in mediating the cardiovascular effects of khat in humans (53). Therefore, the present results allow the suggestion that the analgesic effect of khat is not mediated by direct stimulation of α_1_ receptors and most probably through mechanisms involving noradrenaline release and stimulation of α_2_ receptors. This warrants further studies using selective α_2_ antagonists.

Newly synthesized muscarinic agonists are proved to be analgesic both *in vitro* and *in vivo* (54). Similarly, the inability of atropine to antagonize the antinociceptive action of khat extract on tested pain signals excluded a direct interaction of khat extract with muscarinic receptors. It is reported that the anticholinesterase compound physostigmine attenuated the painful stimuli in formalin test (55). Khat extract was found to inhibit acetylcholinesterase enzyme in khat-chewing individuals (56). Recently, amphetamine and its derivatives are reported to inhibit acetylcholinesterase activity in rats (57, 58). If khat analgesia followed the same scenario, it is conceivable that khat by inhibiting cholinesterase enzyme produced accumulation of acetylcholine, which exerts an antinociceptive action. This explains why the cholinergic antagonist atropine that act on the receptor level did not antagonize the antinociceptive action of khat. Neuronal nicotinic receptors also showed analgesic effects in experimental and clinical trials (59). Whether the antinociceptive effect of khat is mediated through the activation of nicotinic receptors caused by accumulated acetylcholine needs further investigations.

## Conclusion

Khat extract proved to be analgesic against thermic and chemical noxious stimuli in different pain models. The opioidergic and dopaminergic pathways are involved in the antinociceptive action of khat probably by stimulating Aδ fibers. The analgesic effect of khat is also mediated *via* GABA_A_ receptors. The antinociceptive action of khat is not involving direct interaction with α_1_ receptors or the muscarinic cholinergic receptors. Further pharmacological techniques such as binding studies and electrophysiological procedures may be useful to fully elucidate the antinociceptive effects of khat extract.

## Author Contributions

EA put research design, performed data analysis, and writing of the manuscript. HA, AA, and LK shared the idea, experimental design, and manuscript preparation. HA conducted experiments and participated in data analysis and manuscript preparation. All the authors have read and approved the final manuscript for publication.

## Conflict of Interest Statement

The authors declare that the research was conducted in the absence of any commercial or financial relationships that could be construed as a potential conflict of interest.

## References

[1] Johannes CB Le TK Zhou X Johnston JA Dworkin RH. The prevalence of chronic pain in United States adults: results of an internet-based survey. J Pain (2010) 11(11):1230–9. 10.1016/j.jpain.2010.07.002 20797916

[2] Christie MJ. Cellular neuroadaptations to chronic opioids: tolerance, withdrawal and addiction. Br J Pharmacol (2008) 154(2):384–96. 10.1038/bjp.2008.100 18414400 PMC2442443

[3] Soeken KL Miller SA Ernst E. Herbal medicines for the treatment of rheumatoid arthritis: a systematic review. Rheumatology (2003) 42(5):652–9. 10.1093/rheumatology/keg183 12709541

[4] Mossie A Kebedez S Gobena T. Association between khat chewing and intestinal parasitic infestations: a community based, cross-sectional study done in Jimma Town, Southwest Ethiopia. Ethiop Med J (2013) 51:187–95. 24669675

[5] Patel NB. Mechanism of action of cathinone: the active ingredient of khat (*Catha edulis*). East Afr Med J (2000) 77:329–32. 12858935 10.4314/eamj.v77i6.46651

[6] Kebede D Alem A Mitike G Enquselassie F Berhane F Abebe Y Khat and alcohol use and risky sex behaviour among in-school and out-of-school youth in Ethiopia. BMC Public Health (2005) 5:109. 10.1186/1471-2458-5-109 16225665 PMC1274331

[7] Hoffman R Al’Absi M. Khat use and neurobehavioral functions: suggestions for future studies. J Ethnopharmacol (2010) 132:554–63. 10.1016/j.jep.2010.05.033 20553832 PMC2976806

[8] Kalix P Khat, an amphetamine-like stimulant. J Psychoactive Drugs (1994) 26(1):69–74. 10.1080/02791072.1994.10472604 7913130

[9] Zyoud SH Bibliometric analysis on global *Catha edulis* (khat) research production during the period of 1952–2014. Zyoud Globalization Health (2015) 11:39 10.1186/s12992-015-0124-x 26337534 PMC4558725

[10] Nutt D King LA Saulsbury W Blakemore C. Development of a rational scale to assess the harm of drugs of potential misuse. Lancet (2007) 369(9566):1047–53. 10.1016/S0140-6736(07)60464-4 17382831

[11] Feyissa AM Kelly JP. A review of the neuropharmacological properties of khat. Prog Neuropsychopharmacol Biol Psychiatry (2008) 32:1147–66. 10.1016/j.pnpbp.2007.12.033 18561890

[12] WHO. WHO Expert Committee on Drug Dependence. (Vol. 942). Geneva: World Health Organization (2006). p. 1–21. 17373571

[13] Al-Habori M Al-Mamary M. Long-term feeding effects of *Catha edulis* leaves on blood constituents in animals. Phytomedicine (2004) 11(7):639–44. 10.1016/j.phymed.2003.06.004 15636178

[14] Murdoch C Aziz HA Fang HY Jezan H Musaid R Muthana M. Khat (*Catha edulis*) alters the phenotype and anti-microbial activity of peripheral blood mononuclear cells. J Ethnopharmacol (2011) 138(3):780–7. 10.1016/j.jep.2011.10.030 22063724

[15] Nencini P Abdullahi MA Anania MC Moscucci M Paroli E. Prolonged analgesia induced by cathinone. The role of stress and opioid and nonopioid mechanisms. Pharmacology (1984) 29:269–81. 10.1159/000138023 6093160

[16] Nencini P Ahmed AM Naloxone-reversible antinociceptive activity of cathinone, the active principle of khat, in the mouse and rat. Pharmacol Res Commun (1982) 14:759–70. 10.1016/S0031-6989(82)80082-9 7146059

[17] Connor J Makonnen E Rostom A. Comparison of analgesic effects of khat (*Catha edulis* Forsk.) extract, d-amphetamine and ibuprofen in mice. J Pharm Pharmacol (2000) 52:107–10. 10.1211/0022357001773580 10716611

[18] Clarke PB Franklin KB Infusion of 6-hydroxydopamine into nucleus accumbens abolish the analgesic effect of amphetamine but not of MOR in the formalin test. Brain Res (1992) 580:106–10. 10.1016/0006-8993(92)90932-Y 1504789

[19] Świeboda P Filip R Prystupa A Drozd M. Assessment of pain: types, mechanism and treatment. Ann Agric Environ Med (2013) 1:2–7. 25000833

[20] Dickenson A Sullivan A Peripheral origins and central modulation of subcutaneous formalin-induced activity of rat dorsal horn neurons. Neurosci Lett (1987) 83:207–11. 10.1016/0304-3940(87)90242-4 3441298

[21] Grichnik K Ferrante F. The difference between acute and chronic pain. Mt Sinai J Med (1991) 58:217–20. 1875958

[22] Afify E Khedr M Omar A Nasser S. The involvement of K(ATP) channels in morphine-induced antinociception and hepatic oxidative stress in acute and inflammatory pain in rats. Fundam Clin Pharmacol (2013) 27:623–31. 10.1111/fcp.12004 23033987

[23] Hemnani TJ Khan IM Patki VP Dashputra PG Effect of diphenyl-hydantoin with diazepam on electoseizure and chemoseizure susceptibility in mice. Indian J Med Res (1983) 77:521–4. 6874042

[24] Aziz HA Peh KK Tan YT. Extraction and microencapsulation of khat: effects on sexual motivation and estradiol level in female rats. J Sex Med (2009) 6:682–95. 10.1111/j.1743-6109.2008.01157.x 19143913

[25] Nyongesa AW Patel NB Onyango DW Odongo HO Wango EO. Khat (*Catha edulis*) lowers plasma luteinizing hormone (LH) and testosterone secretion, but increases cortisol levels in male rabbits. J Ethnopharmacol (2008) 116:245–50. 10.1016/j.jep.2007.11.022 18180121

[26] Kimani ST Nyongesa AW. Effects of single daily khat (*Catha edulis*) extract on spatial learning and memory in CBA mice. Behav Brain Res (2008) 195:192–7. 10.1016/j.bbr.2008.05.022 18588917

[27] Koster R Anderson M De Beer EJ Acetic acid for analgesic screening. Fed Proc (1959) 18:412–6.

[28] Hunskaar S Hole K. The formalin test in mice: dissociation between inflammatory and non-inflammatory pain. Pain (1987) 30:103–14. 10.1016/0304-3959(87)90088-1 3614974

[29] Woolfe G MacDonald AD The evaluation of the analgesic action of pethidine hydrochloride. J Pharmacol Exp Ther (1944) 80:300–7.

[30] D’Amour FE Smith DL A method for determining loss of pain sensation. J Pharmacol Exp Ther (1941) 72:174–9.

[31] Abdulwaheb M Makonnen E Debella A Abebe D Effect of *Catha edulis* foresk (khat) extracts on male rat sexual behaviour. J Ethnopharmacol (2007) 110:250–6. 10.1016/j.jep.2006.09.019 17079105

[32] Omar AS Modulation of visceral nociception, inflammation and gastric mucosal injury by cinnarazine. Drug Target Insights (2007) 2:29–38. 21901060 PMC3155229

[33] Della Bella D Carenzi A Frigeni V Reggiani A Zambon A. Involvement of monoaminergic and peptidergic components in cathinone-induced analgesia. Eur J Pharmacol (1985) 114:231–4. 10.1016/0014-2999(85)90633-8 4043227

[34] Nencini P Fraioli S Pascucci T Nucerito CV (−)-Norpseudoephedrine, a metabolite of cathinone with amphetamine-like stimulus properties, enhances the analgesic and rate decreasing effects of morphine, but inhibits its discriminative properties. Behav Brain Res (1998) 92:11–20. 10.1016/S0166-4328(97)00123-X 9588681

[35] Lu Y Sweitzer SM Laurito CE Yeomans DC. Differential opioid inhibition of C- and A delta- fiber mediated thermonociception after stimulation of the nucleus raphe magnus. Anesth Analg (2004) 98(2):414–9. 10.1213/01.ANE.0000094334.12027.06 14742380

[36] Chung KM Song DK Huh SO Kim YH Cho MR Suh HW. Supraspinal NMDA and non-NMDA receptors are differentially involved in the production of antinociception by morphine and beta-endorphin administered intracerebroventricularly in the formalin pain model. Neuropeptides (2000) 34:158–66. 10.1054/npep.2000.0805 11021975

[37] Damaj M. Behavioral modulation of neuronal calcium/calmodulin-dependent protein kinase II activity: differential effects on nicotine-induced spinal and supraspinal antinociception in mice. Biochem Pharmacol (2007) 74:1247–52. 10.1016/j.bcp.2007.07.008 17850767 PMC2683468

[38] Al-Awdi S Al-Kadi H Shehab M Effect of khat-habituation on GABA level in brain. Asian J Pharm Life Sci (2013) 3:74–80.

[39] Rodríguez-Landa JF García-Ríos RI Cueto-Escobedo J Bernal-Morales B Contreras CM. Participation of GABAA chloride channels in the anxiolytic-like effects of a fatty acid mixture. Biomed Res Int (2013) 2013:121794. 10.1155/2013/121794 24163810 PMC3791597

[40] Schechter MD Rats become acutely tolerant to cathine after amphetamine or cathinone administration. Psychopharmacology (1990) 101:126–31. 10.1007/BF02253729 1971444

[41] Schechter MD Dopaminergic nature of acute cathine tolerance. Pharmacol Biochem Behav (1990) 36:817–20. 10.1016/0091-3057(90)90083-T 1977178

[42] Svokos K Nalwalk JW Leurs R Menge WM Timmerman HH Hough H. A role for spinal, but not supraspinal, alpha(2) adrenergic receptors in the actions of improgan, a powerful, non-opioid analgesic. Brain Res (2001) 923:12–9. 11743967 10.1016/s0006-8993(01)03191-2

[43] Wang QA Cao JL Zeng YM Ti-jun DA. GABAA receptor partially mediated propofol-induced hyperalgesia at superspinal level and analgesia at spinal cord level in rats. Acta Pharmacol Sin (2004) 12:1619–25. 15569406

[44] Kalix P. Hypermotility of the amphetamine type induced by a constituent of khat leaves. Br J Pharmacol (1980) 68:11–3. 10.1111/j.1476-5381.1980.tb10690.x 7357134 PMC2044110

[45] Banjaw MY Mayerhofer A Schmidt WJ. Anticataleptic activity of cathinone and MDMA (Ecstasy) upon acute and subchronic administration in rat. Synapse (2003) 49:232–8. 10.1002/syn.10236 12827642

[46] Zelger JL Carlini EA. Anorexigenic effects of two amines obtained from *Catha edulis* forsk. (Khat) in rats. Pharmacol Biochem Behav (1980) 12:701–5. 10.1016/0091-3057(80)90152-5 7393964

[47] Kalix P. Pharmacological properties of the stimulant khat. Pharmacol Ther (1990) 48:397–416. 10.1016/0163-7258(90)90057-9 1982180

[48] Banjaw MY Schmidt WJ. Behavioural sensitisation following repeated intermittent oral administration of *Catha edulis* in rats. Behav Brain Res (2005) 156:181–9. 10.1016/j.bbr.2004.05.020 15582104

[49] Banjaw MY Miczek K Schmidt WJ. Repeated *Catha edulis* oral administration enhances the baseline aggressive behavior in isolated rats. J Neural Transm (2006) 113:543–56. 10.1007/s00702-005-0356-7 16082505

[50] Murphy N Lam H Maidment N. A comparison of morphine-induced locomotor activity and mesolimbic dopamine release in C57BL6, 129Sv and DBA2 mice. J Neurochem (2001) 79:626–35. 10.1046/j.1471-4159.2001.00599.x 11701766

[51] Pan HL Wu ZZ Zhou HY Chen SR Zhang HM Li DP. Modulation of pain transmission by G-protein-coupled receptors. Pharmacol Ther (2008) 117:141–61. 10.1016/j.pharmthera.2007.09.003 17959251 PMC2965406

[52] Al-Hashem FH Dallak MA Nwoye LO Bin-Jaliah IM Al-Amri HS Rezk MH Acute exposure to *Catha edulis* depresses contractility and induces myocardial infarction in spontaneously contracting, isolated rabbit’s heart. Saudi J Biol Sci (2012) 19:93–101. 10.1016/j.sjbs.2011.01.002 23961167 PMC3730562

[53] Hassan NA Gunaid AA El-Khally FM Al-Noami MY Murray-Lyon IM. Khat chewing and arterial blood pressure. A randomized controlled clinical trial of alpha-1 and selective beta-1 adrenoceptor blockade. Saudi Med J (2005) 2:537–41. 15900355

[54] Matera C Flammini L Quadri M Vivo V Ballabeni V Holzgrabe U Bis(ammonio) alkane-type agonists of muscarinic acetylcholine receptors: synthesis, in vitro functional characterization, and in vivo evaluation of their analgesic activity. Eur J Med Chem (2014) 75:222–32. 10.1016/j.ejmech.2014.01.032 24534538

[55] Mojtahedin A Tamaddonfard E Zanbouri A. Role of central muscarinic cholinergic receptors in the formalin-induced pain in rats. Indian J Pharmacol (2009) 41:144–7. 10.4103/0253-7613.55205 20442824 PMC2861817

[56] Al-Akwa A Shaher M Al-Akwa S Aleryani S. Free radicals are present in human serum of *Catha edulis* forsk (Khat) abusers. J Ethnopharmacol (2009) 125:471–3. 10.1016/j.jep.2009.07.012 19619634

[57] Rezin GT Scaini G Ferreira GK Cardoso MR Gonçalves CL Constantino LS Inhibition of acetylcholinesterase activity in brain and behavioral analysis in adult rats after chronic administration of fenproporex. Metab Brain Dis (2012) 27:453–8. 10.1007/s11011-012-9331-9 22832793

[58] Varela RB Valvassori SS Lopes-Borges J Fraga DB Resende WR Arent CO Evaluation of acetylcholinesterase in an animal model of mania induced by d-amphetamine. Psychiatry Res (2013) 209:229–34. 10.1016/j.psychres.2012.11.021 23245536

[59] Umana IC Daniele CA McGehee DS Neuronal nicotinic receptors as analgesic targets: it’s a winding road. Biochem Pharmacol (2013) 86:1208–14. 10.1016/j.bcp.2013.08.001 23948066 PMC4127197

